# Circular RNAs and cervical cancer: friends or foes? A landscape on circRNA-mediated regulation of key signaling pathways involved in the onset and progression of HPV-related cervical neoplasms

**DOI:** 10.1186/s12964-024-01494-0

**Published:** 2024-02-10

**Authors:** Emad Heydarnia, Zahra Dorostgou, Neda Hedayati, Vahide Mousavi, Sheida Yahyazadeh, Mina Alimohammadi, Mobina Gheibi, Parasta Heidari, Somayeh Igder, Alireza Mafi, Omid Vakili

**Affiliations:** 1 Department of Medical Nanotechnology, Faculty of Advanced Technologies in Medicine, Iran University of Medical Sciences, Tehran, Iran; 2 Department of Biochemistry, Neyshabur Branch, Islamic Azad University, Neyshabur, Iran; 3 School of Medicine, Iran University of Medical Sciences, Tehran, Iran; 4 School of Medicine, Bushehr University of Medical Sciences, Bushehr, Iran; 5 Department of Immunology, School of Medicine, Shiraz University of Medical Sciences, Shiraz, Iran; 6 Student Research Committee, Department of Immunology, School of Medicine, Shahid Beheshti University of Medical Sciences, Tehran, Iran; 7 Student Research Committee, Mazandaran University of Medical Sciences, Sari, Iran; 8 Department of Clinical Biochemistry, School of Medicine, Ahvaz Jundishapur University of Medical Sciences, Ahvaz, Iran; 9 Department of Clinical Biochemistry, School of Pharmacy and Pharmaceutical Sciences, Isfahan University of Medical Sciences, Isfahan, Iran; 10 Nutrition and Food Security Research Center, Isfahan University of Medical Sciences, Isfahan, Iran; 11 Autophagy Research Center, Department of Clinical Biochemistry, School of Medicine, Shiraz University of Medical Sciences, Shiraz, Iran

**Keywords:** Uterine cervical neoplasms, Human papilloma viruses, Circular RNA, Signal transduction, Diagnosis, Therapeutics

## Abstract

Cervical cancer (CC) is a common gynecologic malignancy, accounting for a significant proportion of women death worldwide. Human papillomavirus (HPV) infection is one of the major etiological causes leading to CC onset; however, genetic, and epigenetic factors are also responsible for disease expansion. Circular RNAs (circRNAs), which are known as a particular subset of non-coding RNA (ncRNA) superfamily, with covalently closed loop structures, have been reported to be involved in the progression of diverse diseases, especially neoplasms. In this framework, abnormally expressed circRNAs are in strong correlation with CC pathogenesis through regulating substantial signaling pathways. Also, these RNA molecules can be considered as promising biomarkers and therapeutic targets for CC diagnosis/prognosis and treatment, respectively. Herein, we first review key molecular mechanisms, including Wnt/β-catenin, MAPK, and PI3K/Akt/mTOR signaling pathways, as well as angiogenesis and metastasis, by which circRNAs interfere with CC development. Then, diagnostic, prognostic, and therapeutic potentials of these ncRNA molecules will be highlighted in depth.

## Introduction

Although cervical cancer (CC) is the fourth commonest malignancy among female population, it can be prevented if diagnosed timely, and then cured efficiently [1, 2]. In the context of this malignancy, the uterine cervix-lining epithelium is the main site, being affected by cervical tumors, in which the squamocolumnar junctions of both ecto- and endo-cervices are considered as the site of choice for carcinomatous onset and being targeted by the malignant cells [3, 4]. It has been estimated that a significant number of CC cases are developed due to human papillomavirus (HPV) infections, as the most prevalent sexually transmitted disease (STD) in the world [5–7]. Squamous cell carcinoma (SCC), as well as adenocarcinoma (AC) are the principal histological species of cervical epithelial neoplasms. Within this context, SCC comprises 95 % of all CCs, whereas the other subtype, i.e. AC, is considered to be responsible for only 5 % of epithelial tumors within the cervix [8, 9]. CCs are mostly treated by conventional anti-tumor therapeutics, namely chemotherapy and radiotherapeutic proceedings but in most cases, these strategies do not result in desirable outcomes and may cause a variety of adverse effects. Thereby, finding novel diagnostic biomarkers along with promising therapeutic targets will open up new avenues in determining the presence and then eradication of tumor cells inside the cervical tissues [10–12].

Noncoding RNAs (ncRNAs), as a great segment of the transcriptome with no significant protein-coding capacity, have been recognized to serve particular modulatory roles during cancer progression, and thus may serve as therapeutic targets to boost survival status in CC patients [13–15]. Approximately, 99 % of the whole RNA in mammalian cells is comprised of non-coding transcripts [16]. According to their functions, ncRNAs can be classified into two subclasses: Housekeeping ncRNAs and regulatory ncRNAs; housekeeping ncRNAs are known for their role in the regulation of general cellular functions, including rRNA modifications, mRNA splicing, and translation [17, 18]. Whilst, regulatory ncRNAs, which are subdivided into short non-coding transcripts (with less than 200 nucleotides (nt.) in length) and long non-coding RNAs (with more than 200 nt. in length) [19], play substantial roles in a variety of biological processes from DNA transcription to mRNA post-transcriptional processing and translation [20–22]. Long non-coding RNAs (lncRNAs), microRNAs (miRNAs), and circular RNA (circRNAs) are the best studied regulatory ncRNAs to date [13, 23–28].

Among the above-stated ncRNA transcripts, circRNAs are of great significance for their structural characteristics that have made them resistant to exonucleases. Indeed, these ncRNAs have covalently closed-loop structures without 5ʹ-to-3ʹ polarity [29, 30]. CircRNAs are largely distributed in multiple tissues, cell types, and even biological fluids [31]. Moreover, they can be packaged into exosomes as well as the other extracellular vesicles (EVs), providing a mechanism for their intercellular communications [32, 33]. Hence, circRNAs can be easily detected and targeted in biological environments within the human body.

The corresponding circular molecules have prominent modulatory effects on cancer-related processes, such as tumorigenesis, tumor progression, apoptosis, etc. [34–36]. Besides, researchers have reported a strong correlation between the expression of circRNAs and the onset of various neoplasms, suggesting that circRNAs may serve as tumor-suppressing and/or promoting molecules [37, 38]. In this framework, the contribution of circRNAs to CC cell proliferation, migration, invasion, and apoptosis has been experimentally evaluated, proposing a hypothesis that declares circRNAs can be considered promising therapeutic targets to fight CC [39, 40]. A thorough understanding of circRNAs and their roles in CC-associated signaling pathways and molecular mechanisms may result in clarification of more powerful therapeutic approaches against CC. Therefore, the current study aims to review the major molecular mechanisms and signaling cascades regulated by circRNAs during the progression of cervical neoplasms, especially those being correlated with HPV infections.

## Pathophysiology of cervical cancer

**Fig. 1:**
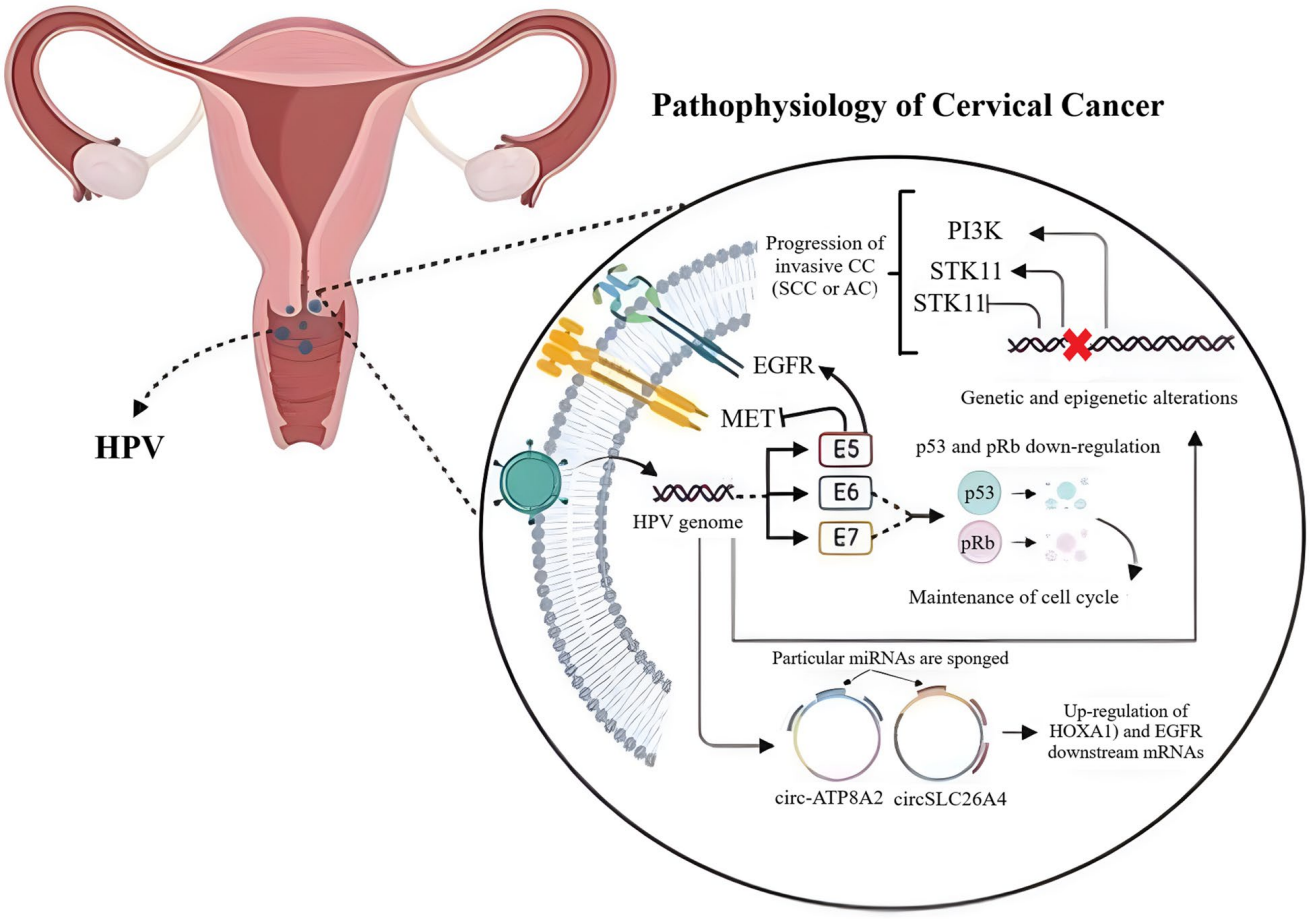
Pathophysiology of cervical cancer. Although genetic mutations and epigenetic alterations can give rise to the onset of CC, HPV infection is also considered a major leading cause for the progression of this gynecological malignancy. Circ-ATP8A2 and circSLC26A4 are key circRNAs, being involved in CC pathogenesis by up-regulating HOXA1 and EGFR mRNAs through sponging specific miRNAs. The figure has been created by https://www.BioRender.com

Although the onset of CC can be controlled by a variety of genetic mutations, as well as epigenetic alterations, its sometimes develops due to a prolonged HPV infection (Fig. 1) [41]. Once HPV-related infection is extended, viral oncogenes are overexpressed, leading to the culmination of complex molecular changes inside the cervical epithelial cells. HPV-encoded onco-proteins, i.e. E6 and E7, are responsible for the modulation of transformation process during the infection-to-cancer transition. The corresponding onco-proteins not only down-regulate major tumor suppressors, including p53 and pRb, but also interact with a vast array of signaling molecules, accounting for the maintenance of cell cycle, as well as genomic stability [42, 43]. In line with E6 and E7 proteins, E5, the other HPV-associated onco-protein, contributes to the processes of invasion and metastasis as it can up-regulate the epidermal growth factor receptor (EGFR) and mesenchymal-epithelial transition factor (c-Met), which is a tyrosine-protein kinase essential for the expression of HPV-related genes [44, 45]. When genetic and epigenetic alterations are gradually augmented inside the infected cells, CC begins to be developed in an undesirable manner. Scanning the mutation patterns through the CC has revealed the presence of non-synonymous somatic nucleotide modifications in diverse genes, such as phosphatidylinositol-4,5-bisphosphate 3-kinase catalytic subunit alpha (PIK3CA), phosphatase and tensin homolog (PTEN), p53, serine/threonine kinase 11 (STK11), and Kirsten rat sarcoma viral oncogene homolog (KRAS) [46–49], among which PIK3CA- and p53-related modifications have greatly been found in squamous cell carcinomas [50]. Clinically, HPV often results in the progression of invasive CCs, either SCC or AC [51]. A significant proportion of SCC cases has been found with pre-determined SILs, which are known as squamous intraepithelial lesions caused by HPV infection. Thus, HPV-related CCs can efficiently be prevented by screening this viral infection via detection of viral nucleic acids [52]. Upon identifying distinct gene expression patterns between SCC and AC, researchers embarked on an investigation to uncover the signaling pathways implicated in these differentially expressed genes (DEGs). To achieve this, they employed Webgestalt, a comprehensive pathway analysis tool, and tapped into three prominent databases, including KEGG, WikiPathways, and Reactome, to gain a global perspective on the deregulation observed in both subtypes. It is crucial to note that each database harbors a unique set of manually curated genes, potentially leading to varied outcomes across the platforms. The KEGG database analysis revealed that the cytokine-cytokine receptor interaction, estrogen signaling, and IL-17 signaling pathways harbored the highest number of deregulated genes. Intriguingly, the JAK-STAT signaling pathway stood out as significantly enriched in this analysis. More interestingly, it was realized that SCC tumors had overactivated IL-17, JAK-STAT, and Ras, than ADC tumors [53].

NcRNAs-correlated epigenetic alterations, including the deregulation of miRNAs, lncRNAs, and circRNAs, have been demonstrated to be responsible for cellular transformations at various stages of cervical intraepithelial neoplastic changes, and thereby the CC development [54–56]. Thus, it can be concluded that circRNAs, along with a defined spectrum of other ncRNAs, significantly participates in the occurrence and development of CC [40]. In this framework, Zhao et al. demonstrated that HPVs had the ability of producing circRNAs, affecting tumor cell growth in vitro and in tumor xenografts [57]. Furthermore, circRNA-miRNA-mRNA regulatory axes were recognized to modulate CC oncogenesis. There are many circRNAs, including circSLC26A4 and circ-ATP8A2, which provoke the onset of CC by up-modulating downstream mRNAs, such as homeobox A1 (HOXA1) and EGFR, mostly by sponging specific miRNAs [58, 59]; the current study will discuss these prominent interactions in detail.

## Circular RNAs at a glance

**Fig. 2:**
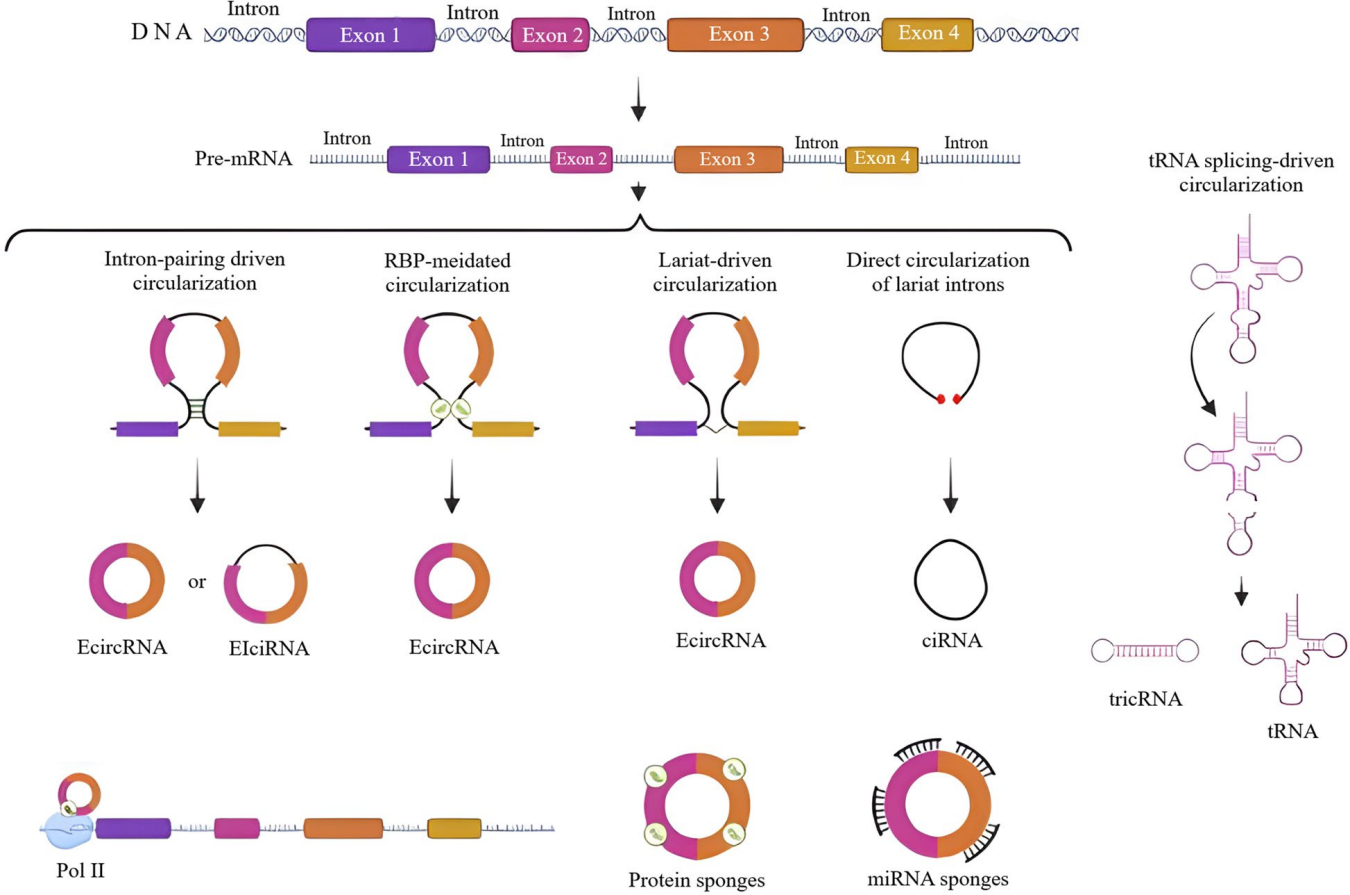
Biogenesis of circRNAs. Pre-mRNA transcripts can undergo (**a**) intron-pairing driven circularization, **b** RBP-mediated circularization, **c** lariat-driven circularization, **d** direct circularization of lariat introns, and/or **e** tRNA splicing-driven circularization to produce circRNAs. EIciRNAs, ecircRNAs, and ciRNAs are the major circRNAs generated by the mentioned circularization methods. The newly produced circRNAs principally serve as protein and miRNA sponges to regulate diverse molecular mechanisms and cellular processes. The figure has been created by https://www.BioRender.com

CircRNAs, as well-studied members of the ncRNAs superfamily, mostly exist in the cytoplasm of eukaryotic cells. Back-splicing, in which a covalent link is formed between a downstream splice-donor region and an upstream splice-acceptor site, is a principal process that conducts the production of circRNAs (Fig. 2) [60–62]. During splicing, circRNAs and their corresponding linear isoforms take part in a competition for biogenesis [38, 63]. The produced circRNAs may then enhance the expression of both circRNAs and mRNAs [64]. Besides, there are particular circRNAs that potentially control the expression of proteins by sequestrating the initiation sites of mRNA translation [65]. Owing to their circular closed-loop structure, circRNAs are not simply degraded by exonucleases [66, 67]; a characteristic that has converted these ncRNA transcripts into high efficient biomarkers for screening diverse defects. A great proportion of these RNA molecules have been realized to serve as competitive endogenous RNAs (ceRNAs) that enter a binding competition with miRNAs through a particular process, called sponging [68]. CircRNAs can also interact with RNA-binding proteins (RBPs) to regulate transcription and splicing, as well as protein translation [69].

According to an approved classification, there are four different categories of circRNAs; as the commonest subclass, exonic circRNAs (ecircRNAs) are originated from exons [70, 71]; on the other front, ciRNAs, as the second subset, are generated from introns [72, 73]; EIciRNAs arise from both exons and introns [64]; and at last, tricRNAs are derived from transfer RNA (tRNA) introns [74]. In the context of circRNA biogenesis, six biogenic models have been identified to date, including: (i) Direct circularization of lariat introns, (ii) intron pairing‐mediated circularization, (iii) lariat‐driven circularization, (iv) RBP‐mediated circularization, and (v) tRNA splicing‐driven circularization [73, 75, 76]. Interestingly, circRNA-producing genes have been found with higher transcription rates compared to other genes that do not generate circRNAs [77]. The production of circRNAs can be triggered by a set of regulatory factors or binding sites, called *cis*-acting elements [78]. Also, this process is under the regulation of RBPs, viz. the fused in sarcoma (FUS), SR protein, heterogeneous nuclear ribonucleoprotein (hnRNP), quaking (QKI), etc. Notably, RBPs can modulate the biogenesis of circRNAs, either positively or negatively [79–82].

The newly generated circRNAs exert several biological functions at various levels of vita. As the best studied functions, they can control gene expression, as well as sponging miRNAs and RBPs, and modulating transcription and translation processes [61, 64, 83, 84]. The direct contribution of circRNAs to gene expression regulation, has made these RNA molecules a prominent group of transcriptional/post-transcriptional modulators [64, 73, 85]. Next, circRNAs are known as miRNA sponges as they compete for miRNA binding sites to restrict their modulatory functions [83, 85]. In this regard, circRNAs have been realized to be more effective than linear miRNA sponges [86, 87]. EIciRNAs and ciRNAs are markedly expressed in the nucleus and have binding sites to target miRNAs. Concurrent with the knock down of these circRNAs, their parental genes will be under-expressed [64, 73]. In relation to RBPs, circRNAs principally function as scaffolds for these proteins to modulate the process of transcription [88, 89]. About the latter function, i.e. translation modulation, despite the non-coding nature of circRNAs, a group of them have surprisingly been found to possess the ability of being translated into functional peptides [90–93].

Recently, a wide spectrum of circRNAs have been identified to be over- or under-expressed through multiple neoplasms, such as CCs. Herein, the subsequent section will focus on CC-related expression pattern attributed to these circular RNA transcripts.

## The expression patterns of circular RNAs during cervical cancer

Using massive parallel sequencing (MPS) and bioinformatics tools, several differentially expressed circRNAs have been detected in CC tissues compared to the non-cancerous counterparts, confirming the significant participation of these RNA molecules in CC (Table 1) [40]. The aberrant expression of circRNAs, observed in CC cells and tissues has been suggested to result in tumorigenesis through sequestering specific miRNAs [94]. The overexpression of these tumor-promoting circRNAs in CC tissues and cells has been linked to shorter survival rates [95]. Within this context, hsa_circ_0141539, a.k.a. circRNA-000284, which is transcribed from the *Clorf116* gene, was found to be overexpressed in CC tissues compared to the adjacent non-tumor counterparts. This circRNA has a direct association with tumor size, FIGO (the International Federation of Gynecology and Obstetrics) classification, and invasion of the myometrium [96]. Hsa_circ_0141539 is a wonderful miR-518d-5p/519-5p sponge, enhancing the expression of chromobox 8 (*CBX8)* gene to accelerate the malignant transformation of cervical cells [96]. Moreover, the circRNA of interest is a miR-506 sponge as well, up-modulating the Snail family transcriptional repressor 2 (*SNAI-2)*, as a direct target of miR-506 [97]. Thus, blocking the hsa_circ_0141539 has been promisingly considered to be a new therapeutic approach for possible cure of CC patients.

**Table 1:** The crosstalk between circRNAs and cervical cancer progression with a focus on signaling pathways and miRNAs that are sponged

**CircRNA**	**Oncogenic/tumor-suppressive function**	**Expression pattern**	**Target miRNA**	**Target signaling pathway**	**Biological roles in CC**	**Reference**
**CircRNF121**	Oncogenic	Overexpression	miR-153-3p	Wnt/β-catenin signaling	CircRNF121 enhances cell proliferation and migration and suppresses autophagy through the miR-153-3p/ATF2 axis and wnt/β-catenin pathway	[98]
**CircSAMD11**	Oncogenic	Overexpression	miR-503	Wnt/β-catenin signaling	CircSAMD11 enhances CC progression, cell proliferation, migration, and invasion and inhibits cell apoptosis by regulating the wnt/β-catenin pathway through the miR-503/SOX4 axis	[99]
**Hsa_circ_0003204**	Oncogenic	Overexpression	_	MAPK signaling pathway	Hsa_circ_0003204 enhances cell proliferation and migration, and suppresses cell apoptosis via regulating the MAPK signaling	[100]
**CircFAT1**	Oncogenic	Overexpression	miR-409-3p	MAPK signaling pathway	CircFAT1 increases tumor growth and CC progression by enhancing the ERK1/2 and p38 of MAPK pathway through the miR-409-3p/CDK8 axis	[101]
**Hsa_circ_0000515**	Oncogenic	Overexpression	miR-326	MAPK signaling pathway	Has_circ_0000515 induces cell proliferation and invasion, while represses apoptosis and autophagy by up-regulating the ELK1 through targeting miR-326	[102]
**Hsa_circ_CSPP1**	Oncogenic	Overexpression	miR-361-5p	PI3K/Akt/mTOR signaling pathway	Hsa_circ_CSPP1 improves proliferation and invasion of CC through miR-361-5p/ITGB1 in PI3K-Akt signaling pathway	[103]
**Hsa_circ_0001627**	Oncogenic	Overexpression	miR-1225-5p	PI3K/Akt/mTOR signaling pathway	Hsa_circ_0001627 promotes CC progression via regulating the hsa_circ_0001627/miR-1225-5p/FNDC3B axis through the PI3K/mTOR pathway	[104]
**Hsa_circ_0001495**	Oncogenic	Overexpression	miR-526b-3p	PI3K/Akt/mTOR signaling pathway	Has_circ_0001495 increases cell proliferation and tumor growth, while decreases apoptosis by upmodulating the TMBIM6 through sponging miR-526-3p	[105]
**CircCCDC134**	Oncogenic	Overexpression	miR-503-5p	_	ALKBH5-mediated m6A modification of circCCDC134 leads to circCCDC134 overexpression, which enhances the CC progression and metastasis by promoting the HIF1A-induced angiogenesis	[106]
**Circ_0006646**	Oncogenic	Overexpression	miR-758-3p	_	Circ_0006646 improves CC progression and metastasis by enhancing the expression of RRM2 by sponging miR-758-3p	[107]
**Circ_0001823**	Oncogenic	Overexpression	miR-613	_	Circ_0001823 promotes cell viability and invasion leading to improved metastasis of CC by increasing the expression of RAB8A through sponging miR-613	[108]
**Hsa_circ_0005358**	Tumor suppressive	Under-expression	_	_	Hsa_circ_0005358 suppresses CC progression and metastasis by blocking the interaction between PTBP1 and CDCP1 and decreasing CDCP1 expression	[109]
**CircLMO1**	Tumor suppressive	Under-expression	miR-4291	_	CircLMO1 enhances CC metastasis by inducing ferroptosis and apoptosis of CC cells through sponging miR-4291	[110]
**CircVPRBP**	Tumor suppressive	Under-expression	miR-106b-5p	_	CircVPRBP suppresses CC metastasis and progression through the miR-106b-5p/TRIM3 axis	[111]
**CircPVT1 (Hsa_circ_0009143)**	Oncogenic	Overexpression	miR-1286	_	CircPVT1 stimulates migration and invasion, and thus pulmonary metastasis. Also, it promotes EMT by sponging miR-1286, in an exosome-dependent manner	[112]
**Circ-0000745**	Oncogenic	Overexpression	_	_	Circ-0000745 upregulation leads to poor differentiation and vascular/lymphatic invasion	[113]
**Circ-000284**	Oncogenic	Overexpression	miR-506	_	Circ-000284 induces tumor growth and invasion by sponging miR-506, and subsequent regulation of Snail-2	[97]
**Circ-NRIP1**	Oncogenic	Overexpression	miR-629-3p	_	Circ-NRIP1 triggers the migration and invasion by inhibiting the PTP4A1/ ERK1/2 through sponging miR-629-3p	[114]
**Circ-0003204**	Oncogenic	Overexpression	_	MAPK signaling pathway	Circ-0003204 induces tumor growth, migration, and invasion by regulating the MAPK signaling	[100]
**CircUBAP2**	Oncogenic	Overexpression	miR-361-3p	_	CircUBAP2 provokes tumor growth and metastasis by targeting the miR-361-3p/SOX4 axis	[115]
**Hsa_circRNA_0001400**	Oncogenic	Overexpression	miR-326	PI3K-Akt signaling pathway	Hsa_circRNA_0001400 improves CC cell proliferation and decreases apoptosis by enhancing the PI3K-Akt signaling pathway	[116]
**Circ_0000263**	Oncogenic	Overexpression	miR-1179	_	Circ_0000263 enhances CC progression by reducing cell apoptosis and amplifying cell proliferation through the miR-1179/ABL2 axis	[117]
**CircRNA_0000285**	Oncogenic	Overexpression	miR-654-3p	_	CircRNA_0000285 suppresses miR-654-3p expression leading to increased cell viability, reduced cell apoptosis, and suppressed CC progression	[118]
**Hsa_circ_0001038**	Oncogenic	Overexpression	miR-337-3p	_	Has_circ_0001038 induces tumor growth, migration, lymph node and myometrial invasion, while suppresses apoptosis by affecting MACC1 and CNNM3 in a miR-337-3p-dependent manner	[119]
**Circ_0003221**	Oncogenic	Overexpression	miR‑139‑3p	_	Circ_0003221 promotes CC angiogenesis and progression via positive enhancing of S100A14 expression through sponging miR-139-3p	[120]

The circRNA hsa_circ_0023404, with an origin of ring finger protein 121 (*RNF121*) gene located on chromosome 11, was the other circRNA found to be up-modulated in CC [121]. Hsa_circ_0023404 can target miR-136 to induce the overexpression of the transcription factor cellular promoter 2 (*TFCP2*) gene, for further activation of the Yes-associated protein (YAP) signaling pathway and subsequent progression of CC [121]. Guo et al. noticed that hsa_circ_0023404 had the ability of sponging miR-5047, as well; by targeting miR-5047, the corresponding circRNA up-modulated the *VEGFA* to accelerate CC metastasis and chemo-resistant trait. Consistently, both hsa_circ_0023404 and *VEGFA* were realized to be concurrently overexpressed in cervical tumors, whereas miR-5047 was notably down-regulated [122].

CircCLK3, which is responsible for enhancing the cell proliferation, migration, and invasion, exerts its oncogenic effects by sponging miR-320a and subsequent stimulation of forkhead box protein M1 (*FoxM1*) gene expression [123]. Has_circ_0000263 was the other overexpressed circRNA detected in CC cells, which consequently affected the p53 at its gene expression levels [124].

Considering the significant role of HPV infection in the onset and deterioration of HPV-related CCs, researchers understood that HPV high-risk strains could encode a specific E7-containng circRNA, namely circE7 [57]. As a viral circRNA, circE7 undergoes N6-methyladenosine (m6A)-induced modifications to be localized inside the cytoplasm, and then being associated with polysomes to independently generate E7 oncoprotein at the end of the story. Thereby, once circE7 is deregulated, E7 oncoprotein will be decreased, leading to the inhibition of tumor growth [57], while its up-regulation turns on signaling pathways, which finally result in tumor cell invasion and metastasis. These findings have shown a circRNA-related molecular mechanism involved in HPV-induced tumorigenesis, suggesting the potential of identifying new viral nucleic acids to differentiate between progressing and regressing cervical lesions.

Together, the expression levels of particular circRNAs have been determined to be altered during CC. These RNA molecules are considered substantial modulators of the CC progression, which serve their functions through regulating a variety of signaling pathways and molecular mechanisms. The best studied circRNA-signaling pathways axes are subsequently discussed in depth.

## How circular RNAs control cervical cancer progression? A focus on underlying signaling cascades and molecular mechanisms

Recent publications have focused on how circRNAs modulate cell signaling pathways as the consequence of miRNA sponging. In this context, circRNAs in relation to vital intracellular pathways have converted into potential targets in cancer therapy. Herein, we discuss the most recent studies on the crosstalk between circRNAs and major signaling pathways through the CC development, which their outcomes have been summarized in Table 1. Eventually, we will also provide beneficial information about the clinical applications of circRNAs in CC theranostics.

### Circular RNAs and the Wnt/β-catenin signaling

Wnt/β-catenin signaling pathway, as a highly conserved mechanism, is principally known for its substantial role in cell homeostasis, cell proliferation, differentiation, apoptosis, along with a vast array of other vital cellular processes [125, 126]. Once the pathway is aberrantly activated, stem cell renewal process as well as differentiation is triggered to improve cancer progression. Therefore, many neoplasms are believed to be suppressed and cured by targeting the corresponding mechanisms [127]. Relative to CC, it has been recognized that circRNA molecules can regulate a plenty of subcellular processes through controlling the Wnt/β-catenin pathway [128].

Within this frame of reference, Wang et al. observed that circRNF121 could enhance the malignant phenotype of CC by modulating the Wnt/β-catenin signaling. In vitro suppression of circRNF121 expression has the ability of decreasing cell proliferation and migration, blocking the epithelial-to-mesenchymal transition (EMT), repressing the autophagy flux, principally by down-regulating the autophagy-related proteins (ATGs), achieved by inhibiting the Wnt/β-catenin pathway. Among putative miRNA targets recognized for circRNF121 (incl. miR-153-3p and miR-665), miR-153-3p exhibited the most levels of alterations, as it was over-expressed following the suppression of circRNF121, confirming circRNF121 as a miR-153-3p sponge. Since miR-153-3p under-expression destroys circRNF121 repression-induced inhibitory effects on cell proliferation, it can be concluded that circRNF121 has cell proliferating abilities by down-regulating the miR-153-3p. Further, miR-153-3p can also inhibit the activating transcription factor 2 (ATF2), as a CC-promoting protein. Thus, when circRNF121 is switched off, ATF2 expression will be notably decreased. Together, circRNF121 interacts with the Wnt/β-catenin signaling by regulating the expression of ATF2 by sponging miR-153-3p. In vivo evaluations also verified the tumorigenic effects of circRNF121 trough manipulating the Wnt signaling during CC [98].

CircSAMD11 is another circRNA molecule found to be overexpressed in CC tissues. Concurrent with circSAMD11 suppression, cell proliferation, migration, and invasion are blocked, while the apoptotic flux is triggered. Moreover, circSAMD11 blockade decreases the expression of proteins participated in the Wnt signaling, as well. As a ceRNA, circSAMD11 directly targets the miR-503, which is a cytoplasmic miRNA responsible for inhibition of CC progression. SRY-box transcription factor 4 (SOX4) is a target gene that is up-regulated in CC cells, which its expression is silenced parallel to circSAMD11 down-modulation. On the other front, miR-503 down-regulation can restore the SOX4 expression, demonstrating the inhibitory effect of miR-503 on the expression of this transcription factor gene. Accordingly, Pan et al. realized that circSAMD11 provoked the CC progression by affecting the Wnt/β-catenin pathway through the aforementioned miR-503/SOX4 axis [99]. In terms of mechanism, circSAMD11 had an opposite effect on the expression of miR-503. Numerous studies have verified that miR-503 inhibits tumors by affecting various cellular biological processes. For instance, it was shown that miR-503 up-regulation reduced glioma cell growth and angiogenesis by binding to leucine-rich repeats and immunoglobulin-like domains-containing protein 2 (LRIG2). MiR-503 down-regulation also suppressed cell proliferation and invasion in retinoblastoma by negatively regulating PTPN12. Furthermore, several investigations indicated a significant reduction of miR-503 in CC, and miR-503 overexpression impeded the development of CC by altering the MIR210HG/miR-503-5p/TNF receptor-associated factor 4 (TRAF4) pathway or targeting AKT2. This is consistent with previous observations that miR-503 was markedly lower in CC. Bioinformatics analysis identified the target relationship between miR-503 and SOX4, which means that the SOX4 level was considerably increased in CC, revealing more details about the molecular mechanism of circSAMD11 in CC advancement [99].

### Circular RNAs and the MAPK signaling cascade

The mitogen-activated protein kinase (MAPK) signaling pathway has been found to be central to a variety of cellular processes, from gene expression to cell growth and proliferation [129, 130]. Cell growth-promoting abilities of the MAPK signaling are attributed to its function downstream of multiple growth-factor receptors. Hence, gene mutations inducing MAPK deregulation are strongly correlated with the pathogenesis of diverse neoplasms [131, 132]. During CC, MAPK signaling is tightly under regulation of a vast array of proteins and RNA molecules, among which circRNAs have been determined to have a significant contribution.

Huang et al. were first who found the relationship between a circRNA and MAPK signaling in CC progression. They noticed that hsa_circ_0003204 could trigger CC development by modulating the MAPK pathway. During their exploration for more in-depth outcomes, they found that hsa_circ_0003204 was overexpressed in CC, which its in vitro inhibition declined CC cell proliferation and migration, while stimulated apoptosis. Using the western blot analysis, they also acknowledged that MAPK signaling-related proteins were overexpressed in line with hsa_circ_0003204 up-modulation, demonstrating the stimulatory effect of hsa_circ_0003204 on cell proliferation through regulating the MAPK signaling mechanism. Beyond the in vitro findings, hsa_circ_0003204 overexpression in nude mice models was found to enhance tumor size and CC progression as well [100].

CircFAT1, a.k.a. hsa_circ_0001461, is another circRNA transcript, being involved in cell proliferation regulation, invasion, and migration [133, 134]. Like in many other cancers, circFAT1 is upregulated in CC, and its overexpression is associated with undesirable survival outcomes in patients. Western blot evaluations showed that the knockdown of circFAT1 reduced the phosphorylation and expression of ERK1/2 and p38, as two major proteins involved in the MAPK signaling; thus, circFAT1 can positively regulate the MAPK pathway to enhance cell proliferation. MiR-409-3p in this regard, is the downstream target of circFAT1, which improves CC cell proliferation. The under-expression of miR-409-3p in CC tissue has confirmed its anti-tumor effects. Cyclin-dependent kinase 8 (CDK8) is a serine-threonine kinase identified with oncogenic effects in multiple cancers. In CC, CDK8 has been reported to be up-regulated, while is suppressed in the presence of miR-409-3p. Ectopic overexpression of miR-409-3p decreases the expression of p-p38/p38 and p-ERK1/2/ERK1/2 proteins of the MAPK signaling, which can be reversed by CDK8 overexpression, suggesting an inhibitory effect for miR-409-3p on cell proliferation and migration through blocking the MAPK pathway achieved by CDK8 suppression. In vivo knockdown of CircFAT1 markedly suppresses tumor growth and reduces the expression of CDK8 as well as p-p38/p38 and p-ERK1/2/ERK1/2. Altogether, circFAT1 facilitates the malignant behavior of CC by amplifying MAPK pathway through the miR-409-3p/CDK8 axis [101].

Another study investigated how the circRNA hsa_circ_0000515 and its related mechanism involving miR-326 affected CC. In CC tissues and cells, miR-326 was initially low, while hsa_circ_0000515 and the ETS-like transcription factor 1 (ELK1) were initially high. Patients with CC who had low levels of hsa_circ 0000515 had a better outcome. The levels of hsa_circ_0000515, miR-326, and ELK1 in CC cells were altered by using different mimics, inhibitors, over-expression plasmids, or siRNAs. Silencing hsa_circ_0000515 or increasing miR-326 caused these cancer cells to undergo apoptosis and autophagy, and reduced their invasion and proliferation, as shown by in vitro experiments. ELK1 was boosted by hsa_circ_0000515, which acted as a ceRNA of miR-326, as confirmed by the RIP, RNA pull-down, and dual-luciferase reporter assays. Higher ELK1 expression also resulted in more invasion and proliferation, but less autophagy and apoptosis, in CC cells. The reduced tumor growth by hsa_circ_0000515 silencing was also verified in vivo. Accordingly, hsa_circ_0000515 can enhance tumor growth in CC patients. The study provided evidence for targeting hsa_circ 0000515 as a potential therapy for CC [102].

### Circular RNAs and the PI3K/Akt/mTOR signal transduction mechanism

Phosphoinositide-3 kinase/protein kinase B (PI3K/Akt) signaling pathway is an intracellular kinase cascade, transferring signals into the cell, and is central to the regulation of cell cycle, cell proliferation, and apoptosis. PI3K activation leads to Akt phosphorylation and localization in the cell membrane. Akt then regulates the expression of several downstream targets, including forkhead box O (FOXO) or mammalian target of rapamycin (mTOR) [135, 136]. The over-activation of the PI3K/Akt signaling has an essential contribution to the pathogenesis of diverse neoplasms, such as the corresponding CC [137–139]. We know that circRNAs can affect the expression of intracellular proteins, especially those being involved in the signaling of interest, via pre- or post-transcriptional regulations. As mentioned before, these RNA molecules can function as oncogenes to provoke cancer progression or even tumor suppressors to block further development of cancers.

In the context of CC, Yang et al. showed that hsa_circ_CSPP1 was a novel promoter of CC carcinogenesis and triggered cell proliferation and invasion of CC through the miR-361-5p/ITGB1 (integrin subunit β1) axis in association with PI3K/Akt signaling pathway. To identify proteins linked to the PI3K/Akt signaling, Yang and colleagues used the STRING online tool; the result showed that ITGB1 was a PI3K/Akt-related integrin protein with an increased expression pattern in CC cells. ITGB1 expression was also correlated to the expression of the corresponding circRNA, i.e., hsa_circ_CSPP1 [103]. Indeed, as a member of the integrin family, ITGB1 connects the extracellular matrix (ECM) to the intracellular tyrosine kinase PI3K, leading to the activation of PI3K/Akt signaling [140]. Then, ITGB1-induced over-activation of PI3K/Akt signaling causes an uncontrolled cell proliferation [141]. Further investigations conducted by the same team revealed that ITGB1 expression was regulated by miR-361-3p in CC cells, in a reverse manner, indicating that miR-361-3p could bind to the ITGB1 to suppress its function for further reduction of cell proliferation and invasion. MiR-361-5p, which is known as a miRNA with tumor-suppressing effects [142], was found to be down-regulated in CC with a negative correlation with hsa_circ_CSPP1 expression, as hsa_circ_CSPP1 was considered a miR-361-5p sponge [103]. Overall, regulatory interactions between hsa_circ_CSPP1, miR-361-5p, and ITGB1 result in PI3K/Akt signaling over-activation, which in turn enhances cell proliferation and invasion to promote CC progression [103].

Hsa_circ_0001627 was an overexpressed circRNA in CC tissues realized by the GEO2R analysis. Unfortunately, CC patients with higher levels of hsa_circ_0001627 expression showed a lower survival rate. Small interfering RNA (siRNA)-induced knockdown of hsa_circ_0001627 inhibited malignant behavior of CC and decreased cell proliferation and invasion. In this regard, miR-1225-5p was identified as a direct target for hsa_circ_0001627 to be down-modulated in CC [104]. Fibronectin type III domain containing 3B (FNDC3B) is a transmembrane endoplasmic reticulum protein that has been shown to affect the progression of cancer [143]. Li et al. declared that FNDC3B was located at the downstream of miR-1225-5p, which miR-1225-5p knockdown could increase the expression levels of FNDC3B mRNA. FNDC3B expression was also positively correlated with hsa_circ_0001627. All these findings indicate that hsa_circ_0001627 modulates the FNDC3B expression via sponging miR-1225-5p. Furthermore, the up-regulated FNDC3B enhances cell proliferation and viability by activating the PI3K/mTOR pathway. In contrast, silencing the hsa_circ_0001627 can reduce the expression of proteins related to the PI3K/mTOR pathway and decrease cancer cell proliferation. Ultimately, it can be concluded that hsa_circ_0001627/miR-1225-5p/FNDC3B axis promotes the CC progression through modulation of PI3K/mTOR signaling pathway. In vivo suppression of hsa_circ_0001627 also decreased tumor size and volume, confirming the tumorigenic effects of the corresponding circRNA [104].

Utilizing the quantitative reverse transcriptase – polymerase chain reaction (qRT-PCR), together with Western blot analyses, the expression levels of miR-526b-3p, transmembrane Bax inhibitor motif containing 6 (TMBIM6), and hsa_circ_0001495 were measured in CC tissues and cells. Experimentally, the upregulated expression of hsa_circ_0001495 and TMBIM6, and downregulated expression of miR-526b-3p were observed in CC tissues and cell lines. In vitro, silencing of hsa_circ_0001495 suppressed tumor growth, while knockdown of TMBIM6 and hsa_circ_0001495 increased cell apoptosis and decreased cell proliferation [105].

### Circular RNAs and the metastasis-related pathways

The process of metastasis, which is a crucial criterion for cancer development, is referred to the pathogenic spread of cancerous cells from where they first generated to other parts of the body that is facilitated through the blood or lymphatic system. In most cases, metastasis is considered a leading cause for treatment failure and premature death [144]. There are several molecular events involved in metastasis development, among which Ras mutations are estimated to be central to 50% of metastatic tumors [145]. Like the above stated mechanisms, circRNAs have the ability of regulating tumor metastasis through a variety of subcellular pathways [146].

One of these well-studied circRNAs is circCCDC134 that can promote CC metastasis and progression by sponging miR-503-5p [106]. CircCCDC134 was found to be overexpressed in mice with metastatic CC; it was reported that circCCDC134 knockdown reduced cell proliferation, migration, and invasion in vitro and decreased tumor volume and nodal involvement in vivo. These findings indicated the role of circCCDC134 in promoting CC metastasis and progression. We know the m6A modification as a substantial post-transcriptional regulation that modifies circRNAs’ function and declines their expression [147]. In this era, the AlkB homologue 5 (ALKBH5) is a primary dioxygenase that demethylases m6A-modified RNAs and decreases RNA degradation to enhance RNA stability [148]. Liang et al. declared that circCCDC134 up-regulation in CC was occurred due to the ALKBH5-mediated m6A modification that improved circCCDC134 stability. Beyond, circCCDC134 is a miR-503-5p sponge, which leads to the up-modulation of the myeloblastosis proto-oncogene (MYB) and its downstream target gene, hypoxia-inducible factor 1 alpha (HIF1A). HIF1A is an important angiogenesis modulator that can be induced by circCCDC134 in order to increase tumor metastasis and progression in a close interaction with p65 and miR-503-5p sponging [106].

Circ_0006646 also has stimulatory effects on CC progression and metastasis. Circ_0006646 with its increased expression pattern in CC cells, can trigger CC-related cell proliferation, migration, and invasion. In line with this fact, exosomes derived from CC mice were also detected with enhanced expression levels of circ_0006646 compared to non-CC mice. Further investigation revealed that miR-758-3p can serve as a direct target for circ_0006646. Typically, miR-758-3p is blocked by circ_0006646, while miR-758-3p suppression reverses the inhibitory effects of circ_0006646 knockdown on cell proliferation, resulting in cell proliferation and invasion, suggesting a role for circ_0006646 in cancer progression through inhibiting miR-758-3p. Ribonucleotide reductase regulatory subunit M2 (RRM2), as an enzyme regulating deoxyribonucleotide production, has been realized to have contribution to a variety of cancers, including CC [149–152]. Yu et al. showed that the expression of RRM2 mRNA and protein was affected by miR-758-3p overexpression. Thereby, circ_0006646 has the ability of enhancing RRM2 expression by sponging miR-758-3p. Contrarily, circ_0006646 repression decreases tumor volume and tumor growth in CC mice [107]. In a distinct study conducted by Ji et al., the tumor suppressor miR-613 was found to be inhibited by circ_0001823, giving rise to tumor cell viability and invasion. Once miR-613 is down-regulated, its downstream target, Rab8A is triggered. Rab8A, which is defined as a specific Ras-related protein-encoding gene, is a small GTPase molecule that regulates intracellular trafficking. Rab8A can function as an oncogenic factor to enhance cancer progression and metastasis [153, 154]. Thereby, circ_0001823 enhances the CC progression and metastasis via increasing Rab8A expression through sponging miR-613 [108].

Conversely, hsa_circ_0005358 was found to suppress CC metastasis by sponging an RBP, called polypyrimidine tract binding protein 1 (PTBP1). During CC, hsa_circ_0005358 shows an under-expression and it can decrease metastasis in vivo if becomes up-regulated. The attachment of hsa_circ_0005358 to PTBP1 is facilitated through specific binding sites to block the interaction between PTBP1 and CUB domain-containing protein 1 (CDCP1), as a nuclear protein that regulates cell division. The overexpression of CDCP1 links to anti-apoptotic and oncogenic effects. Cen et al. revealed that CDCP1 was highly expressed in CC and its expression was negatively correlated with hsa_circ_0005358 expression pattern; the suppression of hsa_circ_0005358 might result in CDCP1 overexpression, leading to an increased cell proliferation, migration, and invasion. Furthermore, PTBP1 repression declines CDCP1 mRNA and protein expression levels, as PTBP1 stabilizes the CDCP1 mRNA and minimizes its degradation. Accordingly, hsa_circ_0005358 has the ability of decreasing CC metastasis via blocking PTBP1 and CDCP1 interaction and subsequent CDCP1 under-expression [109].

Ferroptosis, which is defined as the iron-dependent apoptosis and characterized by lipid peroxidase accumulation, principally contributes to the progression of metastasis [155]. It has been demonstrated that metastasis can be decelerated through inducing the corresponding flux, i.e. ferroptosis. Within this context, circLMO1, a.k.a. hsa_circ_0021087, was found to have the potential of interacting with ferroptosis. Indeed, circLMO1 has tumor suppressive effects on CC that exerts them through modulating ferroptosis, as well as apoptosis [110]. Ou et al. realized that circLMO1 increased the expression of ferroptosis-related mRNAs (incl. ACSL4 or PTGS2) to enhance oxidative stress levels. The expression of ACSL4 mRNA can be induced by miR-4291, as an overexpressed miRNA in CC, which can be sponged by circLMO1. Together, circLMO1 blocks the CC progression and metastasis by inducing the ferroptotic flux through inhibiting the miR-4291/ACSL4 axis [110].

Last but not least, circVPRBP (hsa_circ_0065898) is another tumor suppressive circRNA in correlation with CC metastasis. Through CC, circVPRBP is markedly down-regulated due to its role in suppressing the cancer progression. Mechanistically, it was demonstrated that circVPRBP suppressed CC metastasis and progression by regulating the miR-106b-5p/TRIM3 axis [111]. Tripartite motif-containing protein 3 (TRIM3) is a ligase protein involved in cellular signaling, division, and growth. TRIM3 exerts its tumor-suppressing effects by inhibiting cell proliferation that is developed through arresting the cell cycle [156]. Dual-luciferase reporter and RNA immunoprecipitation assays revealed a significant relationship between circVPRBP, miR-106b-5p, and TRIM3, in which circVPRBP inhibited miR-106b-5p that was responsible for the suppression of TRIM3 expression. Collectively, circVPRBP puts the CC progression brakes on by up-modulating the TRIM3 through repressing miR-106b-5p [111].

Further bioinformatics assessments, along with RT-PCR evaluations indicated that hsa_circ_0009143 (circRNA_PVT1) was overexpressed in CC. Once circRNA_PVT1 is silenced, the migration and invasive traits of CC cells are blocked, decelerating the occurrence of pulmonary metastasis. Contrarily, circRNA_PVT1 upregulation results in migration and invasion induction in CC cells, and thus triggering metastasis to the lung tissues. Additionally, this circRNA was realized to provoke EMT in CC cells by sponging miR-1286 in an exosome-dependent manner, which in turn can be considered a new mechanism of CC expansion [112].

Poor differentiation and vascular/lymphatic invasion have been linked to the upregulation of circ-0000745 in CC tissues. When the activity of Circ-0000745 was reduced, it led to a decrease in E-cadherin, a protein that helps cells stick together, and resulted in less invasion and migration of the tumor cells [113]. Likewise, circ-000284 was found to be overactive in CC cells. It was discovered that this circRNA could induce the growth and invasion of CC by absorbing miR-506 and regulating Snail-2, which is a protein involved in cell movement [97]. Another circRNA, circ-NRIP1, has laso been linked to lymphovascular invasion. This circRNA has been found to encourage the migration and invasion of CC by blocking the protein tyrosine phosphatase 4A1 (PTP4A1)/ extracellular signal-regulated protein kinases 1 and 2 (ERK1/2) pathway and absorbing miR-629-3p [114]. In this era, circ-0003204, which was found to be overactived in CC through RNA sequencing, has also been shown to stimulate the growth, migration, and invasion of CC cells by regulating the MAPK pathway [100].

Last but not least, circUBAP2, as a circRNA being involved in a variety of neoplasms, has been found to be overactived in CC. This circRNA was importantly recognized to influence the miR-361-3p/SOX4 axis, thereby promoting tumor growth and metastasis [115]. Together, the understanding of the role of circRNAs in the spread of CC is expanding, and these findings could potentially lead to new therapeutic strategies.

### Circular RNAs and the apoptosis/angiogenesis-associated mechanisms

Apoptosis is a highly regulated mechanism for programmed cell death to eliminate damaged and/or senescent cells [157, 158]. Considering its substantial role in cell development and homeostasis [159, 160], uncontrolled apoptosis can lead to a wide spectrum of disorders due to the progression of DNA damage and mutations responsible for malignant invasion of cancers [161]. Therefore, apoptosis regulation can be utilized to decelerate the process of cancer progression. CircRNA molecules have been shown to participate in apoptosis development. In other words, circRNAs can enhance cancer progression by inhibiting apoptosis or suppress neoplasms by inducing the apoptotic flux. In the case of CC, Cai et al. specifically conducted an RNA sequencing method to explore diverse circRNAs with aberrant expression patterns in this gynecologic malignancy; their results showed a total of 28 different up-regulated plus 20 different down-regulated circRNAs. They used GO bioinformatics analysis, and then selected hsa_circRNA_0001400 among multiple circRNAs involved in cell proliferation and apoptosis pathways. Hsa_circ_0001400, which was overexpressed in CC, reduced cell proliferation concurrent with its down-regulation. It was found that hsa_circ_0001400 was directly bound to miR-326 through specific binding sites to inhibit its functions. MiR-326 typically blocks cell proliferation and improves apoptosis by direct attachment to Akt and inhibiting the PI3K/Akt signaling pathway. Once hsa_circRNA_0001400 is silenced, miR-326 sponging is abolished, leading to the overexpression of miR-326 and its subsequent anti-tumor effects on CC cells [116].

Circ_0000263 is another oncogenic circRNA with aberrantly up-regulated expression levels in CC [124]. The underlying mechanisms by which tumorigenic effects of circ_0000263 are developed, were analyzed by Zhang et al.; they demonstrated that circ_0000263 knockdown significantly reduced cell proliferation and induced cell apoptosis in CC. Blocking the expression of circ_0000263 increased the expression of miR-1179, along with some other miRNAs, which their overexpression were not considerable [117]. MiR-1179 has tumor suppressing effects in multiple cancers [162], and its under-expression in Zhang et al. study confirmed the suppressive role attributed to miR-1179 in CC progression. More interestingly, ABL2 (Abelson murine leukemia viral oncogene homologue 2) that is a tyrosine kinase responsible for the regulation of tumorigenesis and tumor cell growth, was indicated to be repressed by miR-1179. Thus, miR-1179 silencing could significantly increase the expression of ABL2, leading to enhanced cell proliferation and decreased cell apoptosis. Altogether, it was proven that circ_0000263 induced CC progression by decreasing cell apoptosis and increasing cell proliferation through the above stated miR-1179/ABL2 axis [117, 163].

A newly identified circRNA with prominent carcinogenic roles in CC progression, as well as bladder cancer and laryngocarcinoma, is circ_0000285 [164–166]. MiR-654-3p, on the other front, is a miRNA with oncostatic characteristics in a variety of neoplasms, such as colorectal and lung cancers [142, 167]. MiR-654-3p is also known a suitable downstream target for circ_0000285 [168]. Mechanistic evaluations revealed that circ_0000285 was up-regulated in CC and considered a miR-654-3p sponge. SiRNA-induced knockdown of circ_0000285 increased miR-654-3p expression, diminishing cell viability, increasing cell apoptosis, and suppressing the CC progression. Thereby, circ_0000285 down-modulation can desirably be in line with CC deceleration [118].

Relative to apoptosis, hsa_circ_0001038 levels were also found to be altered in CC cell lines and tissues. By utilizing Loss/Gain-of-function assays, it was examined how has_circ_0001038 affected the apoptosis, as well as expansion, invasion, and migration of CC cell lines. This circRNA was observed to be upmodulated in CC cells and tissues, and that clinical severity, including lymph node invasion and myometrial invasion, was correlated with high expression of hsa_circ000038. Also, a poor prognosis for CC patients was linked to hsa_circ_0001038 overexpression. hsa_circ_0001038 was silenced, which resulted in an increase in cell apoptosis but a decrease in cell migration, invasion, and growth. Cell oncogenic properties were enhanced by forced expression of hsa_circ_0001038. Hsa_circ 0001038 may bind to miR-337-3p in order to alleviate its inhibition on metastasis-associated in colon cancer 1 (MACC1) and cyclin-M3 (CNNM3), thereby promoting the growth of CC cells and their potential for invasion, respectively, for the purpose of mechanism exploration. As a result of CNNM3 and MACC1 activation, hsa_circ_0001038 functions as an oncogene in CC cells [119].

In continuation of highly expressed circRNAs in CC, circ_0003221 was found to be a promoter of CC progression, through controlling the process of angiogenesis [151]. In parenthesis, angiogenesis is the formation of blood vessels from the existing vasculature to nutritionally support the newly generated tumor cells. Hence, it can be concluded that angiogenesis suppression may cease the process of cancer development [169]. Chi et al. investigated the role of circ_0003221 in CC development and realized that its down-regulation could restrict cell proliferation and invasion. Interestingly, circ_0003221 knockdown decreases the protein expression levels of VEGFA and fibroblast growth factor 2 (FGF2), indicating the inhibitory effects of circ_0003221 on tumor angiogenesis [151]. Exploring mechanisms of angiogenesis suppression by circ_0003221 revealed that this circRNA exerted its oncogenic effects via inhibiting miR‑139‑3p. MiR‑139‑3p is downregulated during CC and is negatively correlated with circ_0003221 expression. S100 proteins, as a group of calcium-binding proteins with defined carcinogenic effects were identified to be inhibited by miR-139-3p in CC. In this regard, S100A14 is a well-defined tumorigenic S100 protein that is under the regulation of the corresponding miRNA. Cell proliferation and invasion traits have been reported to be suppressed in miR-139-3p-transfected cells, which S100A14 can recover cell proliferation as well as angiogenesis in the same cells. Conclusively, circ_0003221 has the ability of promoting CC angiogenesis and progression by positive regulation of S100A14 expression through targeting the miR-139-3p [120]. Using circRNA microarrays for comparing the expression of circRNAs between the normal and CC cell lines, circNRIP1 (hsa_circ_0004771) was also found here to be markedly expressed in SiHa CC cell line [151]. It has been assumed that this circRNA may be linked to angiogenesis during CC cell growth and expansion; however, the hypothesis has not yet been confirmed, and thus further explorations and analyses are still needed.

## Clinical applications

### Circular RNAs, promising biomarkers for screening and diagnosis of cervical cancer

**Fig. 3:**
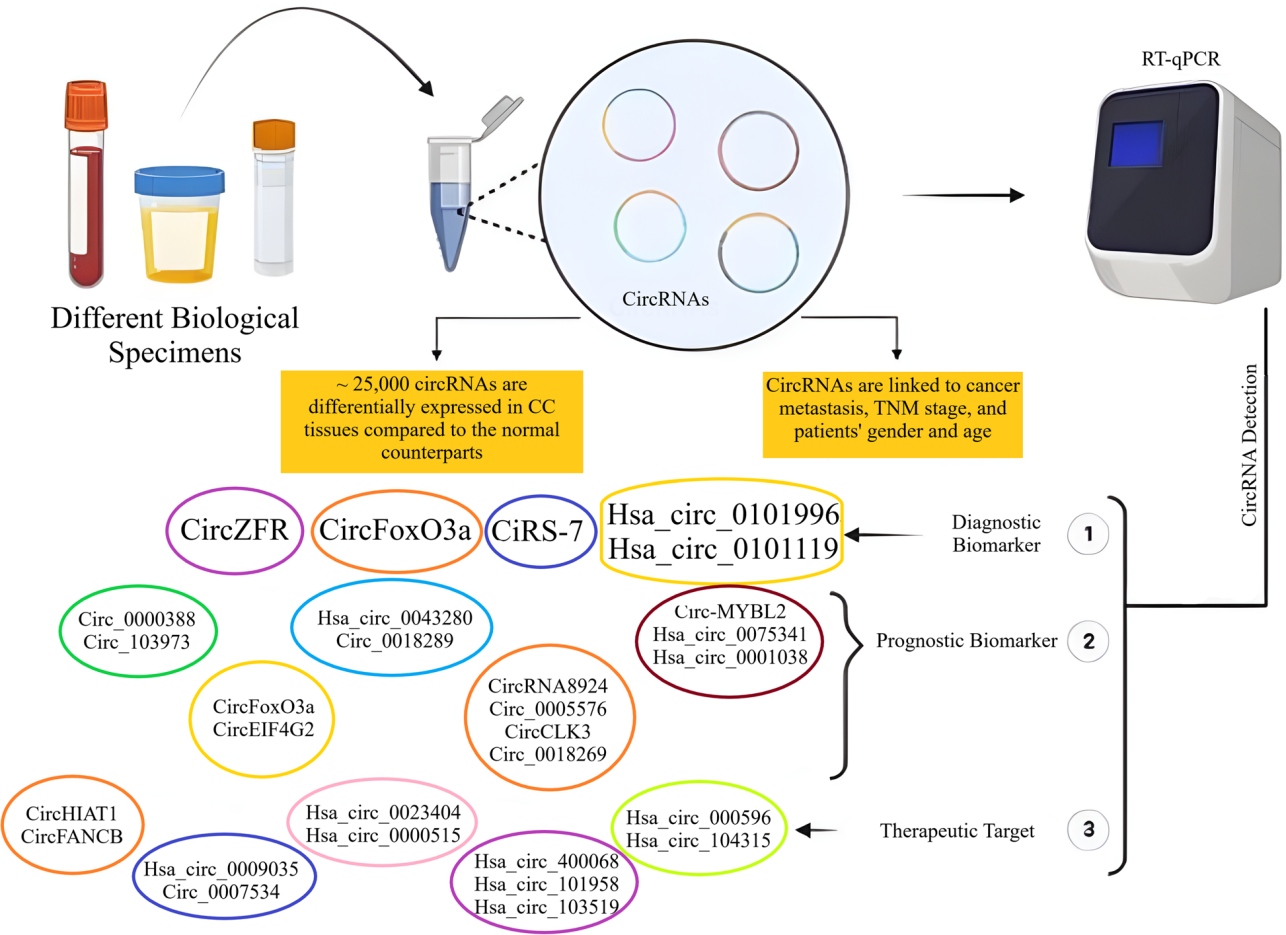
Clinical applications of circRNAs in diagnosis, prognosis, and treatment of cervical cancer

In recent years, liquid biopsies have been found as potential alternatives for the conventional tissue biopsies. This is of great importance when a particular biomarker is easily detected through non-invasive or minimally invasive liquid biopsy-based strategies [94]. Currently, a large number of non-invasive biomarkers, including carcinoembryonic antigen (CEA), carbohydrate antigen 19–9 (CA19-9), and squamous cell carcinoma antigen (SCC Ag), are clinically used to detect and monitor the CC cases by only measurement in whole blood specimens [170]. A vast array of circRNAs have recently been suggested to be helpful in CC detection with acceptable diagnostic thresholds (Fig. 3). In this era, the mechanisms by which circRNAs affect the CC pathophysiology may lead to identification of new biological targets. CircRNAs are tissue specific and have desirable structural stability, making them resistant to exonucleases. These RNA molecules can be easily detected in human blood, as well as saliva, urine, and other body fluids, enhancing the potential of circRNAs to become non-invasive diagnostic biomarkers [171]. Moreover, circRNAs are linked to cancer metastasis, TNM stage, patient gender, and age [172]. Wang and colleagues recognized that almost 80,000 circRNAs were expressed in CC tissues and matched non-cancerous counterparts, of which about 25,000 were differentially expressed. Their findings confirmed reliable evidence for the expression of circRNAs in CC [173, 174]. Hopefully, this potential of circRNAs is not limited to CC and these non-invasive biomarkers have demonstrated potential diagnostic applications for a wide spectrum of human malignancies. The common q RT-PCR is the major confirmatory tool to detect circRNAs in clinical samples [175, 176].

In this framework, Zhou et al. realized that ciRS-7 is in strong correlation with tumor size, advanced FIGO stage, lymph node invasion, HPV infection, and worse prognosis for CC patients. They also showed that this circRNA was more potential in differentiation of CC and CIN (cervical intraepithelial neoplasia) cases form healthy subjects in comparison to routine tumor markers, such as CEA and CA125. Once ciRS-7 is combined with those two tumor markers, the best outcomes will be achieved. In differentiation of CC patients from healthy controls, ciRS-7 showed a sensitivity of 95% along with a specificity of 89%. In the case of CIN differentiation from non-cancerous individuals, a sensitivity of 70% with a specificity of 90% were observed, and lastly, the sensitivity and specificity of ciRS-7 for CC differentiation from CIN were reported 88% and 73%, respectively [34].

Beyond the ciRS-7 expression levels, the whole blood expression profiles of other circRNAs have also been reported with specific changes in CC patients. Hsa_circ_0101996, hsa_circ_0104649, hsa_circ_0104443, and hsa_circ_0101119 were the four circRNAs investigated to be significantly overexpressed in blood specimens collected from CC patients versus the healthy control subjects. Using the Receiver Operating Characteristic (ROC) curve, researchers noticed that hsa_circ_0101996 and hsa_circ_0101119 had the area under the ROC curve (AUC) of 0.906 and 0.887, respectively [177]. CircFoxO3a was another circRNA in this regard that had an inverse correlation with lymph node stromal invasion depth. In CC patients, the under-expression of serum circFoxO3a was in negative association with overall survival (OS) rate [178]. Thereby, hsa_circ_0101996, hsa_circ_0101119, and circFoxO3a isolated from peripheral whole blood of CC patients can be combined for being employed as diagnostic biomarkers in clinical settings. Furthermore, ROC evaluation showed AUCs of 0.907, 0.869, and 0.88 for circ_0018289, hsa_circ_0107593, and circZFR, respectively [179, 180]. Consistently, the pathological features of 40 CC patients demonstrated a positive correlation between circZFR expression and lymphatic metastasis, Ki67 values, and squamous cell carcinoma antigen (SCC Ag) value [181]. Thus, the corresponding circRNAs, i.e. circ_0018289, hsa_circ_0107593, and circZFR can also be considered as screening tools for CC diagnosis.

Together, circRNAs are promising biomarkers with diagnostic applications in CC, just like many other neoplasms; nevertheless, further analyses are still needed to be uses instead of or in combination with the conventional diagnostic approaches.

### Circular RNAs to monitor cervical cancer prognosis

CircRNAs not only control the cell proliferation, migration, invasion, and metastasis of CC cells through modulating diverse signaling mechanisms but also have the ability of predicting the overall survival (OS) rate of patients with recurrence (Fig. 3) [94, 182]. Tissue overexpression of oncogenic circRNAs is in strong association with poor survival rates in patients, while tumor suppressor circRNAs overexpression is related to good prognosis and higher survival rates [39]. Currently, circFoxO3a and circEIF4G2, which are found in CC tissues, have been reported to be in onnection with CC prognosis. Tang *et al*. noticed that patients with squamous CC and low serum circFoxO3a levels had a poorer prognosis [178, 183]. On the other hand, the overexpression of circEIF4G2 was found to be linked to tumor size and lymph node metastasis. By the Kaplan–Meier curve analysis and comparing CC samples, an inverse correlation was revealed between circEIF4G2 expression levels and the patients’ survival rate. Thus, an increase in expression of the corresponding circRNA indicates the poor prognosis of CC patients [173]. Cox regression analysis have also demonstrated that a few number of circRNAs, *viz*. hsa_circ_0043280 and circ_0018289, along with clinicopathological features can serve as independent factors for worse prognosis in CC patients [39].

According to q RT-PCR analyses, the expression levels of circ-MYBL2 [184], hsa_circ_0075341 [185], hsa_circ_0001038, circRNA8924 [96], circ_0005576 [186], circCLK3 [123], and circ_0018289 [179] were found to be significantly up-modulated in CC cell lines and cancerous tissues. Moreover, the overexpression of these circRNAs were reported to be in close interaction with tumor size, FIGO staging, lymph node metastasis, myometrial invasion, and poor prognosis in CC patients. During CC progression, hsa_circ_0075341 can down-regulate miR-149-5p, which its low expression levels are associated with undesirable OS rate [185]. CC patients who exhibit higher expression levels of hsa_circ_0001038, as the other prognostic circRNA, can decrease OS in a more desired manner compared to those with lower expression status. Hsa_circ_0001038 has a negative association with miR-337-3p in CC tissues. The expression of this circRNA is also positively related to the expression levels of metastasis-associated in colon cancer 1 (MACC1) and cyclin-M3 (CNNM3). Indeed, hsa_circ_0001038 permits miR-337-3p to release the blockade of CNNM3 and MACC1 for being associated with metastasis to provoke the growth and invasion of CC cells [119]. In addition, the up-regulation of circ_0000388 in CC clinical specimens links to poor pathological indicators. Once circ_0000388 is overexpressed, miR-337-3p expression is hindered, and then TCF12 expression is increased to exert its tumorigenic effects on CC cells and tissues [187]. Circ_103973 overexpression is also an indicator of poor prognosis in CC patients [188]. Ultimately, circ_101996 was identified to be positively associated with CC staging and negatively related to OS rate [189].

All these findings demonstrate that circRNAs can be employed as potential prognostic markers in CC cases. High-throughput RNA sequencing methods have facilitated the exploration of circRNA transcripts with prognostic potentials in cancerous patients versus healthy individuals; notwithstanding, it is necessary to prepare the circumstances for circRNAs entering clinical settings in the near future.

### Circular RNAs as therapeutic targets to combat cervical cancer

Surgery and chemotherapy are the conventional therapeutic strategies against CCs [190, 191]. There are a large number of chemo drugs for treating the patients diagnosed with CC, among which cisplatin has been introduced as the commonest agent; however, the majority of these medicines have been reported to face chemo-resistance [192]. It has been reported that some circRNAs may be involved in cancer cell resistance against common chemotherapeutics. For instance, hsa_circ_0023404 has been identified to develop cisplatin resistance through promoting angiogenesis and autophagy [193]. It has recently been demonstrated that baicalein has anti-tumor effects on CC through modulation of circHIAT1 by subsequent inhibition of Akt/mTOR pathway [194]. It was also found that hsa_circ_0009035 knockdown could improve radio-resistance in CC cells through miR889-3p/HOXB7 axis [39]. Silencing of oncogenic circRNAs, such as circ_0007534, can also suppress malignant traits, such as tumorigenesis, in cervical neoplasms [195]. On the contrary, the overexpression of tumor suppressive circRNAs has the same consequences as those reported for oncogenic circRNAs suppression.

Regarding the recent in-depth research on circRNAs, abnormally expressed circRNAs have been proposed to serve as specific therapeutic targets to combat diverse types of CC (Fig. 3). In this context, hsa_circ_0000515, which is a highly expressed circRNA in CC tissues and cells, plays an oncogenic role to promote cell proliferation, migration, and invasion of CC cells [102]. It has been reported that its suppression can inhibit cell growth, invasion, and inducing apoptosis, as well as autophagy. Yue et al. in a distinct study recognized that hsa_circRNA_000596, hsa_circRNA_104315, hsa_circRNA_400068, hsa_circRNA_101958, and hsa_circRNA_103519 might have the ability of competing for endogenous RNA by generating a modulatroy circRNA-miRNA-mRNA network to target RRM2. The aforementioned circRNAs can contribute to the regulation of mRNA rs5030743 and rs1130609, as well as other similar SNP treatments of CC optional chemo-drugs [196]. In line with these circRNAs, in vitro analyses showed that circ_0104541 down-modulation could also block the migration and invasion of CC cells to ameliorate this female-specific neoplasm [186].

Interestingly, Spatholobi Caulis tannin (SCT) is a traditional Chinese medicine with anti-tumor properties against a variety of cancers. SCT can decrease the survival rate of Hela cells and control the cell cycle [197]. Molecular docking evaluations, along with other bioinformatic analyses predicted SCT target proteins for further exploration of circRNAs linked to SCT target genes, and RegRNA analysis finally represented the following projections: CircFANCB by targeting hsa-miR-4692 and circE2F3 though sponging hsa-miR-5006-3p, hsa-miR-3960, hsa-miR-4739, hsa-miR-4459, hsa-miR-211-5p, and hsa-miR-4632 had interactions with SCT target genes. Since SCT can regulate CC-related circRNAs to induce apoptosis and block cell proliferation, can be considered as a therapeutic booster for cervical neoplasms [198].

In the context of clinical utilization of circRNAs, circRNA-based therapeutic strategies have only been tested in preclinical investigations and several obstacles exist that need to be conquered to use these approaches in the clinical setting. Some of these limitations can be listed as off-target gene silencing in non-cancerous environments, targeting non-specific cells or tissues, toxicity of circRNAs-containing nanovesicles, generation of mis-spliced products, and progression of immunogenicity against therapeutic circRNAs [199]. RNA interfering interventions (RNAi) and gene editing are the two most common targeted therapies nowadays. The CRISPR-Cas9 system is a gene editing technique in this regard that specifically removes the Alu sequence, which is essential for circRNA formation. This only affects the formation of circRNA, which regulates the cell’s vital functions, and not the mRNA amount of the gene’s linear product. Notwithstanding, this method often causes unpredictable cutting events and has ethical concerns, as DNA editing is permanent. On the other front, RNAi does not change genes and is a safer method to modify RNA levels inside the cells. It introduces shRNA or siRNA into cells, which cuts circRNA and reduces its levels. Moreover, the CRISPR-Cas13 system has been shown to have a better performance in circRNA knockdown in terms of efficiency and specificity and is increasingly used to target circRNA without interfering with mRNA. However, there are still some problems with low delivery of gRNA and Cas13 enzyme into target cells, which need to be solved before CRISPR-Cas13 technology can be truly applied in clinic [200, 201].

## Conclusions

CircRNAs have been found to affect a variety of biological processes in association with CC progression, including cell proliferation, apoptosis, invasion, and therapeutic resistance. These effects are mostly achieved through regulating molecular mechanisms, as well as substantial signaling cascades. In this regard, some circRNAs have the ability of operating signaling mechanisms, including the PI3K/Akt/mTOR signaling, Wnt/β-catenin pathway, and MAPKs cascade. Furthermore, cancer-related processes, such as angiogenesis and metastasis can also be affected by circRNAs. Beyond the role of circRNAs in CC pathogenesis and expansion, they can be detected in different biological specimens, suggesting diagnostic and therapeutic capacities for these ncRNA transcripts. Although circRNAs are suitable biomarkers to screen CC diagnosis and monitor its prognosis, our current knowledge of this set of ncRNAs are limited, and further evidence is still needed to clarify the exact mechanisms by which circRNAs are linked to CC deterioration.

The discovery of novel non-invasive or minimally invasive circRNA-identifying techniques is boosting the CC circRNA-based diagnostics and therapeutic strategies, which is converting into a milestone in eradicating of this gynecologic neoplasm.

## Data Availability

Not applicable.
